# Supplementary material for: Environmental impact of single-use, reusable, and mixed trocar systems used for laparoscopic cholecystectomies
Source: PLoS One. 2022 Jul 15;17(7):e0271601. doi: 10.1371/journal.pone.0271601 (PMC9286249; doi:10.1371/journal.pone.0271601)
Supplement: S1 Appendix — (PDF) [file pone.0271601.s001.pdf]

S1 Appendix

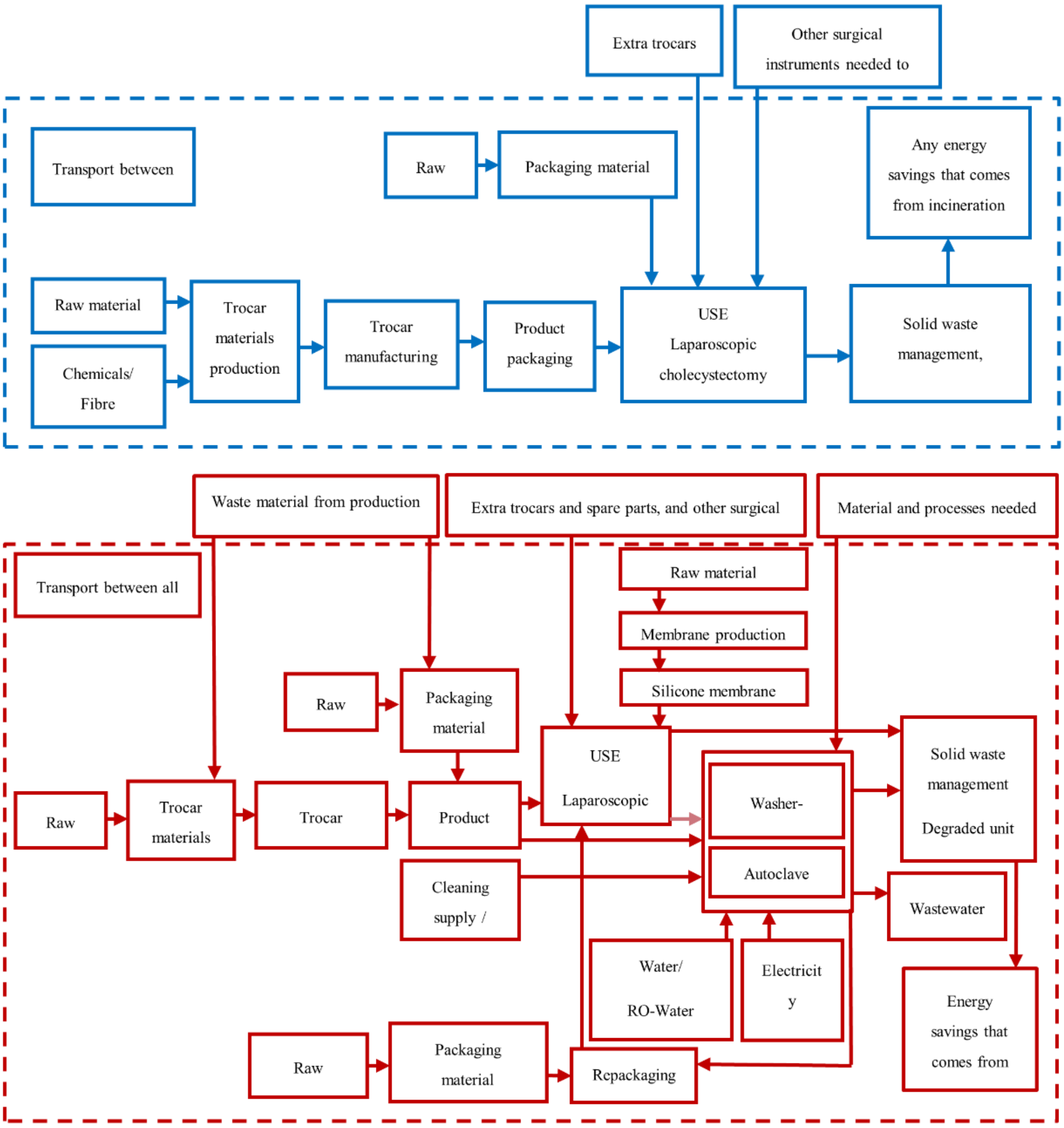

Fig A. System boundaries. Single-use trocar system boundaries (blue). Reusable trocar system boundaries (red). Processes inside the dashed line are included in the system boundaries, processes outside are excluded from the LCA.

Table A. Life cycle inventory data quality

Product system	Material or process	Direct data	Estimated data	Retrieved Online
Single-use system	Trocar material			X
	Trocar weight		X	
	Trocar production processes		X	
	Packaging material and production		X	
	Transport		X	
Reusable system	Trocar materials	X		
	Trocar weight	X		
	Trocar production processes		X	
	Packaging material and production	X		
	Sterilization wrap		X	
	Transport	X		
Mixed system	Trocar materials	X		
	Trocar weight	X	X	
	Trocar production processes		X	
	Packaging material and production	X		
	Sterilization wrap	X		
	Transport	X	X	
Sterilization process	Washer disinfector	X		
	Autoclave	X		

Direct data refers to information provided by supplier or by weighting the products on sight at the hospital. Estimated data refers to estimations based on information on other products. Retrieved online refers to information collected on supplier's website.

Table B. Specification of material and process inputs reusable trocar system

**Trocars Landskrona Hospital**		
**Reusable trocar 10 mm (cannula + obturator)**		
Part	Material and processes	Input in SimaPro
Canula	Not disclosed [a]	20 g carbon fibre reinforced plastic, injection moulded (RER)
		20 g polyvinylchloride, bulk polymerised (GLO)
Obturator		17.3 g ethylene vinyl acetate copolymer (RER)
		62 g steel, chromium steel 18/8 (GLO)
		3.2 g polyvinylchloride, bulk polymerised (GLO)
Processes		62 g metal working, avarage for chromium steel product manufacturing (GLO)
		40.5 g injection moulding (GLO)
**Reusable trocar 5 mm with stopcock (cannula + obturator)**		
Part	Material and processes	Input in SimaPro
Canula	Not disclosed [a]	6.4 g ethylene vinyl acetate copolymer (RER)
		10.1 g carbon fibre reinforced plastic, injection moulded (RER)
		11.1 g brass (ROW)
		0.9 g polyvinylchloride, bulk polymerised (GLO)
Obturator		62 g steel, chromium steel 18/8 (GLO)
		11.1 g brass (ROW)
		10 g ethylene vinyl acetate copolymer (RER)
		0.9 g polyvinylchloride, bulk polymerised (GLO)
Processes		25.3 g injection moulding (GLO)
		23.4 g metal working, avarage for chromium steel product manufacturing (GLO)
		12.2 g metal working, avarage for metal product manufacturing (GLO)
**Reusable trocar 5 mm without stopcock (cannula)**		
Part	Material and processes	Input in SimaPro
Canula	Not disclosed [a]	6.4 g ethylene vinyl acetate copolymer (RER)
		10.1 g carbon fibre reinforced plastic, injection moulded (RER)
		0.9 g polyvinylchloride, bulk polymerised (GLO)
Processes		7.3 g injection moulding (GLO)
		1.65 tkm transport, freight, lorry 16-32 metric ton, euro5 (RER)
		0.28 tkm transport, freight, sea, ferry (GLO)
		500 n sterilization processes

The different parts and weight of the respective trocar used at Landskrona Hospital based on information provided by the manufacturer, Surgical Innovation, Great Britain. Corresponding data from the ecoinvent 3.6 database was used as input in SimaPro for the production material and processes. Input information valid for Europe (RER), rest of the world (ROW), globally (GLO).

[a] On request by the manufacturer.

Table C. Specification of material and process inputs single-use trocar system

**Trocars Lund Hospital**		
**Single-use trocar 12 mm, VersaOne 12 mm bladeless trocar (cannula + obturator)**		
Part	Material an processes	Input in SimaPro
Canula 31 g	Transparent polyamide	9 g glass fibre reinforced plastic, polyamide, injection moulded (GLO)
	Polysoprene (synthetic rubber)	2 g synthetic rubber (GLO)
	C-Flex Thermoplastic	9 g polyurethane, flexible foam (RER)
	Elastomer	2 g synthetic rubber (GLO)
	Marlex 9006 H	9 g polyethylene, high density, granulate (GLO)
Obturator 22 g	Transparent polyamide	8 g glass fibre reinforced plastic, polyamide, injection moulded (GLO)
	Acrylonitrile-butadiene-styrene	7 g acrylonitrile-butadiene-styrene copolymer (GLO)
	Versaflex Thermoplastic Elastomer	7 g polyurethane, flexible foam (RER)
Processes	Plastic processing	36 g injection moulding (GLO)
**Single-use trocar 5 mm, VersaOne 5 mm bladeless trocar (cannula + obturator)**		
Part	Material and processes	Input in SimaPro
Canula 6 g	Transparent polyamide	2 g glass fibre reinforced plastic, polyamide, injection moulded (GLO)
	Polysoprene (synthetic rubber)	0.5 g synthetic rubber (GLO)
	C-Flex Thermoplastic	2 g polyurethane, flexible foam (RER)
	Elastomer	0.5 g synthetic rubber (GLO)
	Marlex 9006 H	1 g polyethylene, high density, granulate (GLO)
Obturator 15 g	Transparent polyamide	5 g glass fibre reinforced plastic, polyamide, injection moulded (GLO)
	Acrylonitrile-butadiene-styrene	5 g acrylonitrile-butadiene-styrene copolymer (GLO)
	Versaflex Thermoplastic Elastomer	5 g polyurethane, flexible foam (RER)
Processes	Platic processing	14 g injection moulding (GLO)
	Transport for 1x12 mm trocar, 1x5 mm trocar, 2x12 mm cannula, 2x5 mm cannula	0.28 tkm transport, freight, lorry 16-32 metric ton, euro5 (RER)
		0.02 tkm transport, freight, sea, ferry (GLO)

The different parts and weight of the respective trocar used at Skåne University Hospital, Lund. Material and process information was gathered from the manufacturer, Medtronic's website (Medtronic) for a similar product. Corresponding data from the ecoinvent 3.6 database was used as input in SimaPro for the production material and processes. Input information valid for Europe (RER), rest of the world (ROW), globally (GLO).

Table D. Specification of material and process inputs mixed trocar system

**Trocars Helsingborg Hospital**		
**Single-use trocar 5-12 mm, FIOS obturator with advanced fixation sleeve (cannula + obturator).**		
Part	Material / Process	Input in SimaPro
40 g cannula	Polyester colorant (polycarbonate, with colorant)	40 g polycarbonate (GLO)
3 g retention disk	Elastomer	3 g synthetic rubber (GLO)
< 1 g trocar sleeve	Adhesive+ Polyolefin	0.5 g polyethylene, low density, granulate (GLO)
21 g obturator	Polycarbonate	21 g polycarbonate (GLO)
4 g "sleeve syringe"	Plastic (unknown)	4 g polyethylene, high density, granulate (GLO)
8 g package holder	Plastic (unknown)	8 g polycarbonate (GLO)
Processes	Plastic processing	76.5 g injection moulding (GLO)
	Transport for 1 trocar	0.09 tkm transport, freight, lorry 16-32 metric ton, euro5 (RER)
**Reusable trocar 10 mm, Trocar Sleeve Slant Cap 10mm standard port (cannula + obturator)**		
Part	Material / Process	Input in SimaPro
86 g cannula	Stainless steel titanium blend	86 g steel, chromium steel 18/8 (GLO)
41 g obturator	Stainless steel titanium blend	41 g steel, chromium steel 18/8 (GLO)
< 1 g membrane	Silicone	0.9 g polyvinylchloride, bulk polymerised (GLO)
<1 g stopcock	Plastic (unknown)	1 g polycarbonate (GLO)
Processes	Plastic processing	1 g injection moulding (GLO)
	Metal processing	127 g metal working, avarage for chromium steel product manufacturing (GLO)
**Reusable trocar 5 mm, Trocar Sleeve Strai. Cap 5.5mm (cannula + obturator)**		
Part	Material / Process	Input in SimaPro
11 g cannula	Plastic (unknown)	11 g polycarbonate (GLO)
27 g obturator	Stainless steel titanium blend	27 g steel, chromium steel 18/8 (GLO)
<1 g membrane	Silicone	0.9 g polyvinylchloride, bulk polymerised (GLO)
Processes	Transport for all 5 + 10 mm reusable trocars	0.67 tkm transport, freight, lorry 16-32 metric ton, euro5 (RER)
	Plastic processing	11 g injection moulding (GLO)
	Metal processing	27 g metal working, avarage for chromium steel product manufacturing (GLO)
	Sterilization process for all reusable trocars	500 sterilization processes

The different parts and weight of the respective trocar used at Helsingborg hospital. Information on the single-use trocar was provided by the manufacturer, Applied Medical, Netherlands. Information on the reusable trocars was provided by the Swedish distributor, Kungshusen AB, Sweden. Corresponding data from the ecoinvent 3.6 database was used as input in SimaPro for the production material and processes. Input information valid for Europe (RER), global (GLO).

Table E. Specification of packaging material and processes

**Trocar packaging material**		
Part	Material and processes	Input in SimaPro
Plastic coverage	Not disclosed [a]	7.5 g polyethylene, high density, granulate (GLO)
Cardboard box		3.5 g solid bleached board (GLO)
Processes		7.5 g extrusion, plastic film (GLO)
Sterilization wrap	48.6 g Polypropylene	39 g polypropylene, granulate (GLO)
	48 g weaving	38.5 g weaving, syntethic fibre (GLO)

The material and weight of the different trocar packages used at Landskrona Hospital, with a specification of material input in SimaPro. Information on plastic coverage and cardboard box was provided by the manufacturer, Surgical Innovation, Great Britain. Information on sterilization wrap was provided by sterile technician at Landskrona Hospital. Corresponding data from the ecoinvent 3.6 database was used as input in SimaPro for the production material and processes. Input information valid globally (GLO).

[a] On request by the manufacturer

Table F. Specification of sterilization material and processes

**Sterilization machines**			
Type of device	Product name	Input in SimaPro	Comment
Washer-disinfector	8668T	110 liter water (SE)	
		30 liter deionized water (RER)	In SimaPro the input of ionized water is provided in weight unit. 1 liter was assumed equal to 1 kg.
		5 ml alkylbenzene sulfonate (GLO)	In SimaPro the input of detergent is provided in weight unit. 1 ml was assumed equal to 0.95 mg.
		6.5 kWh electricity mix (SE)	
Autoclave	GSS76H	228 liter water (SE)	
		15.5 liter deionized water (RER)	
		10.9 kWh electricity mix (SE)	

The total amount of materials and processes needed in the sterilization process for one full autoclave program and one full washer-disinfector program for the machines used at Landskrona Hospital. Information was provided by Getinge AB, Sweden. Input information valid for Sweden (SE), Europe (RER), globally (GLO). Default data from the ecoinvent 3.6 database was used for the production material and processes.

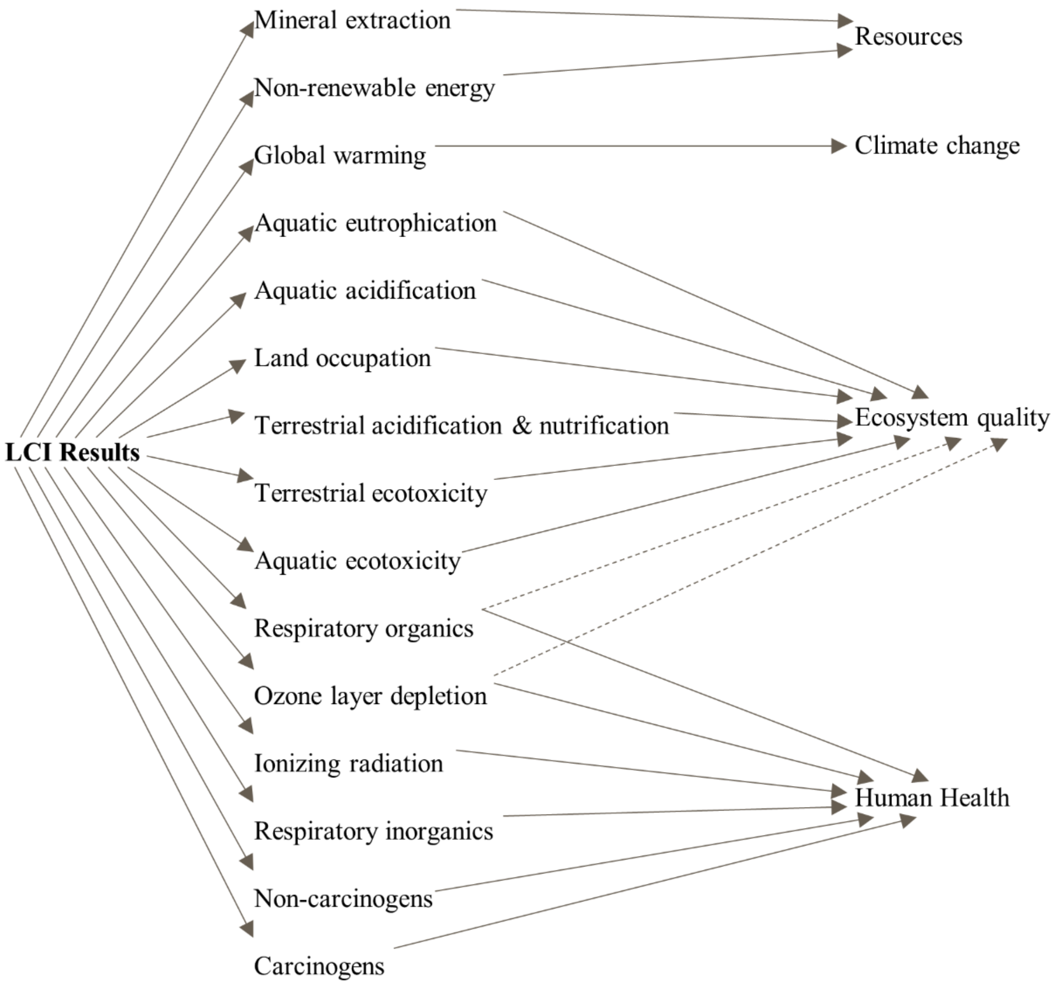

Fig B. Model of Life Cycle Impact Assessment method IMPACT 2002 +. Impact categories and pathways to endpoint categories covered by the IMPACT 2002+ methodology adapted from Jolliet et al. (2003) where dashed lines represent uncertain pathways.

Table G. Specification on costs related to the sterilization process

Material or process	Cost	Included	Excluded	Comment, source of information
Water	0.004 € / liter	X		Average cost for water in Sweden in 2019 (1).
Deionized water	0.25 € / liter	X		Average cost for deionized water in Sweden (2).
Electricity	0.092 € / kWh	X		Average cost for energy in south of sweden in year 2020 (3)
Pickup hazardous waste	125 € / pickup		X	Regardless fraction. It was excluded from the calculation due to the negligible share of total waste amount (4).
Waste treatment, biological wast	1.2 € / kg	X		Cost per kilo for treatment of biological waste (4).
Pickup paper/plastic recycling	78 € / pickup		X	16-22 m^3. It was excluded from the calculation due to the negligible share of total waste amount (5).
Purchase and service autoclave	1432 € (reusable system)	X		The cost is based on the trocars allocated share of a fully loaded program (for reusable system 2 and 8%, for the mixed system 1.5 and 6% of the autoclave and washer-disnifector, respectively). Furter allocation was made based on the number of programs in which the trocars were included troughout the machines' life times (trocars are included in every second program). Total cost for puchase and service of autoclave was 143 200 €. Total cost for purchase and service of washer disinfector was 12 600 €. Information provided by Landskrona Hospital.
	1074 € (mixed system)	X		
Purchase and service washer-disinfector	505 € (reusable system)	X		
	379 € (mixed system)	X		
Labour cost	3800 € (reusable system)	X		Labour cost was based on the avarage time needed to clean one trocar (4 minutes), times the total amount of trocars needed for 500 surgeries (2000 and 1500 respecitivelly for the reusable and mixed system), times the avarage hourly salary, including general payrol taxes, for a laboratory technician (29 €/h) and specialist assistant nurse (28 €/h), assuming that workload was split equally between the two (6, 7).
	2850 € (mixed system)	X		
Sterilization wrap	0.56 € / surgery	X		Cost based on the trocars total share of instrument on the tray (35%). Total cost for a sterilization wrap was 1.6 €.

Specification of costs related to the sterilization process.

1. Dricksvattenfakta.: Svenskt Vatten; 2019 [cited 2021 Mars 16]. Available from: https://www.svensktvatten.se/fakta-om-vatten/dricksvattenfakta/.
2. Persson KM, Berghult B, Elfström-Broo A. Fluoridrening av dricksvatten – en litteraturstudie. Motala: Svenskt Vatten AB; 2003.
3. Energimarknadsinspektionen. Historiska jämförpriser på elhandelsavtal 2021 [cited 2021 Mars 16]. Available from: https://www.ei.se/sv/statistik/statistik-inom-omradet-el/Statistik-om-elhandel/historiska-jamforpriser-pa-elhandelsavtal/.
4. Hantering av farligt avfall, kemikalier och sekretessavfall: Region Skåne; 2020 [Available from: https://contracts.opic.com/Contract/Details/1690851.
5. Avfallshantering för Region Skåne.: Region Skåne; 2021 [cited 2021 Mars 16]. Available from: https://contracts.opic.com/Contract/Details/1690851.
6. Lönestatistik. Undersköterska löner 2020 [cited 2021 May 1]. Available from: https://www.lonestatistik.se/loner.asp/yrke/Underskoterska-1242.
7. Lönestatistik. Steriltekniker löner 2020 [cited 2021 May 1]. Available from: https://www.lonestatistik.se/loner.asp/yrke/Steriltekniker-2690.

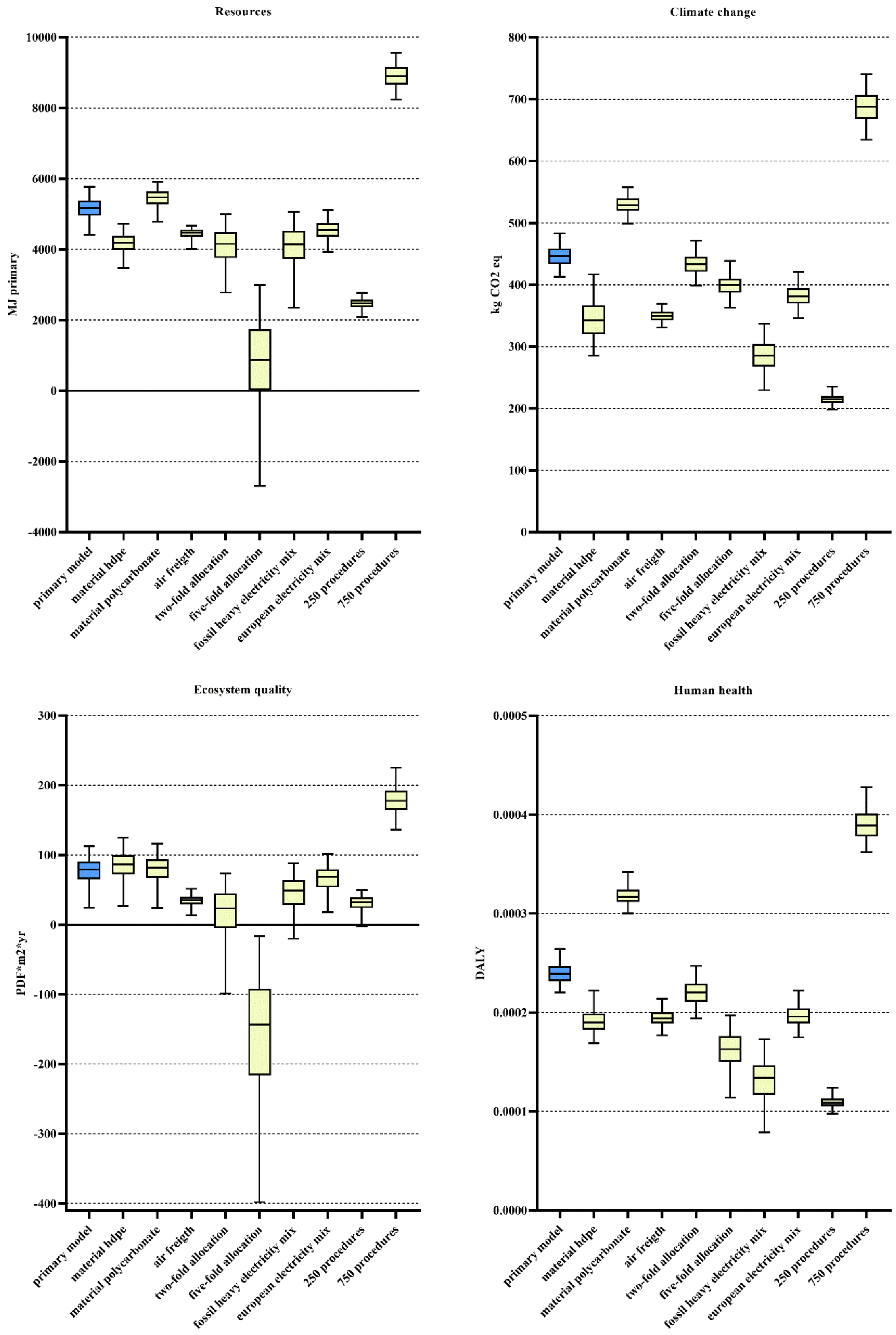

Fig C. Median difference between the single-use and reusable trocar systems' result in the sensitivity analyses. The median differences of primary analysis (blue) compared to sensitivity analyses (yellow) with changes in materials (changing plastic materials to HDPE or Polycarbonate), in

transportation (changing to air freight), in the allocation in the sterilization process (two- or five-fold increase in the autoclave and washer-disinfector's impact), in the electricity mix (changing to European average or fossil fuel heavy), and in the number of procedures (changing to 250 or 750). Data are presented as median and the 2.5th to 97.5th percentiles. There is a difference between the two alternatives if the 2.5th percentile is ≥ 0 or the 97.5th percentile is ≤ 0. A positive value indicate that the single-use system had a higher impact.

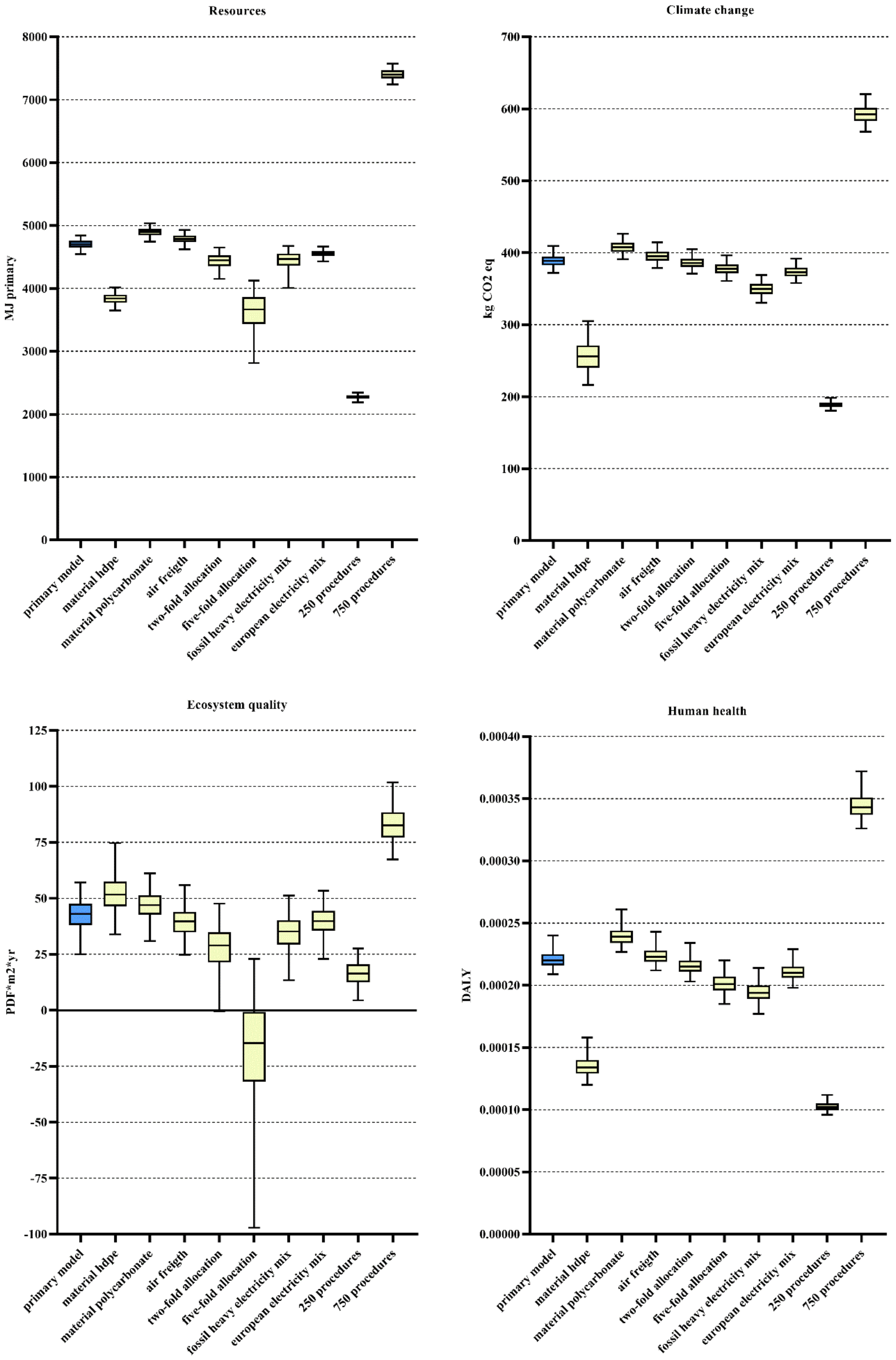

Fig D. **Median difference between the mixed and reusable trocar systems' result in the sensitivity analyses.** The median differences of primary analysis (blue) compared to sensitivity

analyses (yellow) with changes in materials (changing plastic materials to HDPE or Polycarbonate), in transportation (changing to air freight), in the allocation in the sterilization process (two- or five-fold increase in the autoclave and washer-disinfector's impact), electricity mix (changing to European average or fossil fuel heavy), and in the number of procedures (changing to 250 or 750). Data are presented as median and the 2.5th to 97.5th percentiles. There is a difference between the two alternatives if the 2.5th percentile is ≥ 0 or the 97.5th percentile is ≤ 0. A positive value indicate that the mixed system had a higher impact.

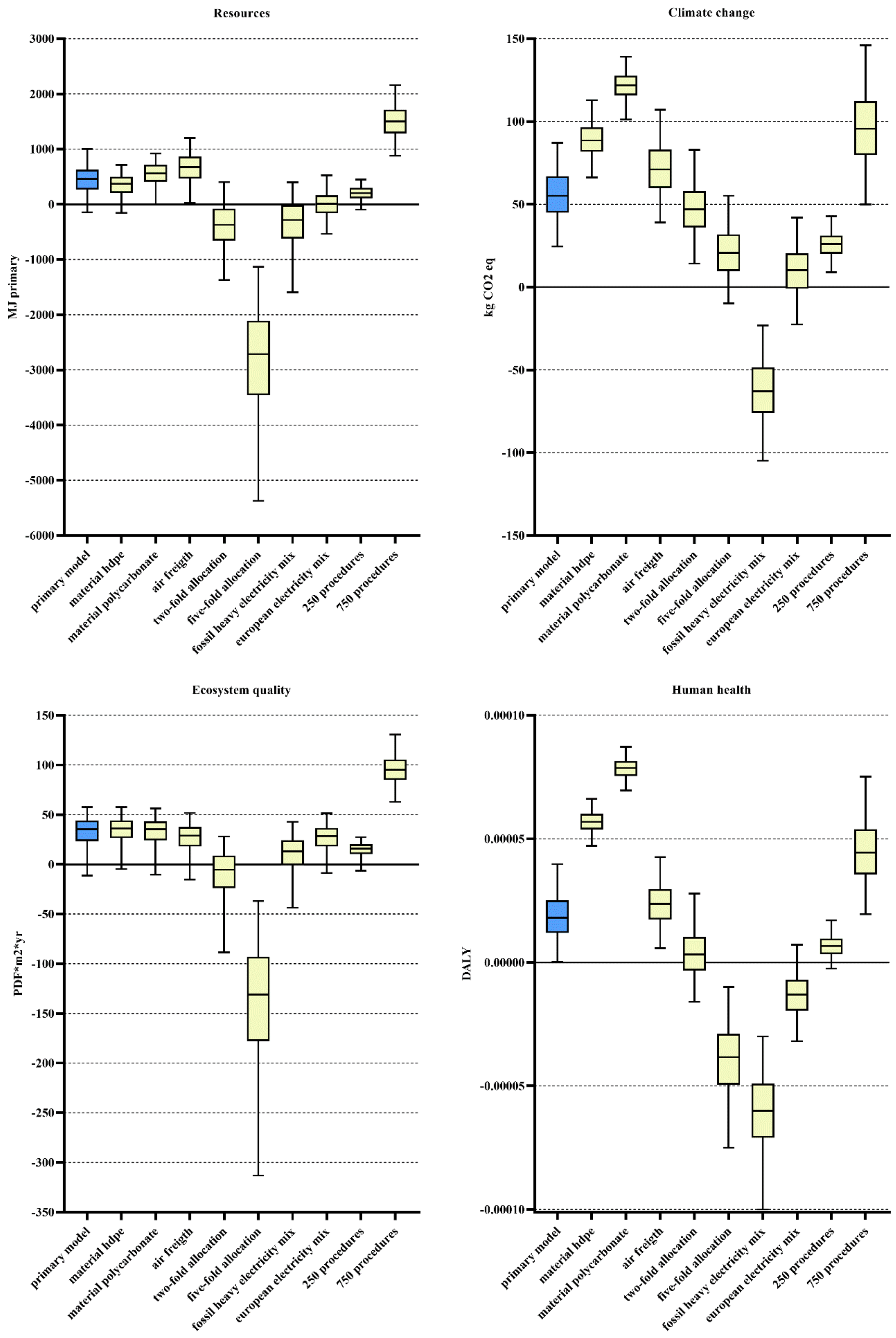

Fig E. Median difference between the single-use and mixed trocar systems' result in the sensitivity analyses. The median differences of primary analysis (blue) compared to sensitivity analyses (yellow) with changes in materials (changing plastic materials to HDPE or Polycarbonate), in transportation

(changing to air freight), in the allocation in the sterilization process (two- or five-fold increase in the autoclave and washer-disinfector's impact), in the electricity mix (changing to European average or fossil fuel heavy), and in the number of procedures (changing to 250 or 750). Data are presented as median and the 2.5th to 97.5th percentiles. There is a difference between the two alternatives if the 2.5th percentile is ≥ 0 or the 97.5th percentile is ≤ 0. A positive value indicate that the single-use system had a higher impact.

Table H. Specification of total cost calculation.

Single-use product system				
**Product/service**	**Unit**	**Unit price**	***Amount***	**Cost**
single-use trocar 5-12 mm	/piece	€ 29.00	500	€ 14,500
single-use trocar 5 mm	/piece	€ 15.00	500	€ 7,500
cannula 5-12 mm	/piece	€ 18.00	500	€ 9,000
cannula 5 mm	/piece	€ 13.00	500	€ 6,500
Treatment biological waste	kg	€ 1.20	55.5	€ 66
Transport [a]	-	-	-	-
**Total cost**				**€ 37,567**

Reusable product system					
**Product/service**	**Unit**	**Unit price**	**Amount**	**Allocation**	**Cost**
Reusable trocar 10-12 mm	/piece	€ 469	2	100%	€ 938
Reusable obturator 10-12 mm	/piece	€ 224	1	100%	€ 224
Reusable trocar 5 mm with stopcock	/piece	€ 268	5	100%	€ 1,340
Reusable trocar 5 mm without stopcock	/piece	€ 201	5	100%	€ 1,005
Reusable obturator 5 mm	/piece	€ 84	5	100%	€ 420
Membrane	/piece	€ 7	1000	100%	€ 7,000
Tap water washer-disinfector	liter	€ 0.004	55000	8%	€ 17
RO water washer-disinfector	liter	€ 0.25	15000	8%	€ 300
Electricity washer-disinfector	kWh	€ 0.09	3250	8%	€ 23
washer-disinfector purchase	/machine	€ 12,600	1	4%	€ 504
Tap water autoclave	liter	€ 0.004	114000	2%	€ 9
RO water autoclave	liter	€ 0.25	7750	2%	€ 38
Electricity autoclave	kWh	€ 0.09	5450	2%	€ 9.81
autoclave purchase and service	/machine	€ 143,000	1	1%	€ 1,430
labor cost sterile technician	/h	€ 29	67	100%	€ 1,943
labor cost specialist assistant nurse	/h	€ 28	67	100%	€ 1,876
Sterilization wrap	/piece	€ 1.60	500	35%	€ 280
Treatment biological waste	Kg	€ 1.20	0.5689	100%	€ 0.68
Transport [a]	-	-	-	-	-
**Total cost**					**€ 17,359.36**

Mixed product system					
**Product/service**	**Unit**	**Unit price**	**Amount**	**Allocation**	**Cost**
Single-use trocar 5-12 mm	/piece	€ 21	500	100%	€ 10,500
Reusable trocar 10 mm	/piece	€ 480	1	100%	€ 480
Reusable obturator 10 mm	/piece	€ 120	1	100%	€ 120
Reusable trocar 5.5 mm	/piece	€ 118	10	100%	€ 1,180
Reusable obturator 5 mm	/piece	€ 65	5	100%	€ 325
membrane	/piece	€ 3.55	290	100%	€ 1,029
Tap water washer-disinfector	liter	€ 0.004	55000	6%	€ 13
RO water washer-disinfector	liter	€ 0.25	15000	6%	€ 225

Electricity washer-disinfector	kWh	€ 0.09	3250	6%	€ 17.5
washer-disinfector purchase	/machine	€ 12,600	1	3%	€ 378
Tap water autoclave	liter	€ 0.004	114000	1.5%	€ 6.84
RO water autoclave	liter	€ 0.25	7750	1.5%	€ 29
Electricity autoclave	kWh	€ 0.09	5450	1.5%	€ 7.36
autoclave purchase and service	/machine	€ 143,000	1	0.75%	€ 1,072
labor cost sterile technician	/h	€ 29	50	100%	€ 1,450
labor cost specialist assistant nurse	/h	€ 28	50	100%	€ 1,400
Sterilization wrap	/piece	€ 1.60	500	35%	€ 280
Treatment biological waste	kg	€ 1.20	38.623	100%	€ 46
Transport [a]	-	-	-	-	-
**Total cost**					**€ 18,560**

The costs included in the respective product systems, purchase price and price for sterilization (if applicable). The total cost is the sum of the trocars and sterilization process for 500 laparoscopic cholecystectomies.

[a] Cost for transport were excluded for all systems as the total cost were below € 0.1, based on fuel prices in May 2021.
