# Supplementary material for: Environmental impact of single-use, reusable, and mixed trocar systems used for laparoscopic cholecystectomies
Source: PLoS One. 2022 Jul 15;17(7):e0271601. doi: 10.1371/journal.pone.0271601 (PMC9286249; doi:10.1371/journal.pone.0271601)
Supplement: Attachment — Submitted filename: Response to Reviewers.docx [file pone.0271601.s002.docx]

Dear Huan Li,

We want to thank the reviewers for the kind words and constructive comments. We have now revised the manuscript to address the concerns of the reviewers and hope that the manuscript can now be accepted for publication in PLOS ONE. Detailed responses to the reviewers are enclosed below.

Response to additional requirements:

1. Please ensure that your manuscript meets PLOS ONE's style requirements, including those for file naming.

**We have checked the file naming and it should now be in line with the style requirements of PLOS ONE. We have also run the figures through the PACE digital diagnostic tool to ensure that the figures meet PLOS requirements.**

2. We note that the grant information you provided in the ‘Funding Information’ and ‘Financial Disclosure’ sections do not match.

**It was our understanding that a financial disclosure was not supposed to be included in the manuscript as advised in the submission guidelines “*Do not include funding sources in the Acknowledgments or anywhere else in the manuscript file. Funding information should only be entered in the financial disclosure section of the submission system”.* We have now included a financial disclosure after the conclusion in the manuscript so that it matches the funding information given in the submission system and we hope that this is satisfactory.**

3. Please review your reference list to ensure that it is complete and correct.

**No retracted paper has been cited. We have however rearranged the reference list after revising the manuscript and the following reference has been added:**

- **McGain, F., McAlister, S., McGavin, A., Story, D. A Life Cycle Assessment of Reusable and Single-Use Central Venous Catheter Insertion Kits, Anesthesia & Analgesia. 2012;114(5): 1073-1080.**

On behalf of the authors,

Linn Boberg

Reviewer: 1

We thank reviewer 1 for the kind words and constructive comments. Below is a response to each of the queries:

1. However, some parts of this paper still should be restructured and described, such as the Abstract and Conclusion.

**Reply: This is a valid comment and we have added additional information in the abstract (introduction/conclusion) and the conclusion which we hope contributes to a clearer background and context for in which the results are valid.**

Reviewer: 2

We thank reviewer 2 for the kind words and constructive comments. Below is a response to each of the queries:

1. The introduction is rather brief, and certain concerns require more explanation. Although "surgical procedures are one of the most resource intensive activities in healthcare" and their environmental effects should be studied, there are various sorts of surgical procedures, and the author should explain why they studied laparoscopic cholecystectomy. Furthermore, related studies must be summarized and reviewed in order to identify research gaps and emphasize the objective and significance of this study.

**Reply: This is valid remarks and we have now rewritten the introduction to clarify the context and rationale for choosing to assess different trocars used for laparoscopic cholecystectomies. We have also included a section with a summary of previous studies which has assessed or compared the environmental impact of reusable and single-use items.**

2. The conclusion is far too brief. In the end, in addition to describing the new results, there should be some enlightenment or application.

The point is, I believe it is true that reuse can lessen environmental impact; nevertheless, whether reuse has any negative consequences, such as economic expenses and potential human health risks, the author must describe in thoroughly, otherwise readers may be misled.

**Reply: This is a valid concern and we have changed the conclusion so that it is clear for which context the results are valid.**

3. Line 60-71, the authors should comment on the findings of the Ref. 12 (Environmental impact and life cycle financial cost of hybrid (reusable/single-use) instruments versus single-use equivalents in laparoscopic cholecystectomy), highlighting the contrasts between the two studies.

**Reply: We have now provided a more detailed rationale for the need of additional studies in the field of laparoscopic surgery and emphasized the need for an assessment of the precision of any differences between the compared alternatives. Such an analysis was not presented in Ref 12. (now Ref. 19). We have now also added a sentence in the discussion in which we emphasize this analysis as an extension of the previous work presented in Ref 12. (now Ref. 19).**

4. Line 104, Table 1. Product summary.

**Reply: Thank you for this comment, this has now been corrected in our manuscript.**

5. Line 188-200, the software (or processes) used to perform the Monte Carlo simulation, as well as the number of iterations required.

**Reply: Thank you for pointing this out, this has now been corrected in our manuscript.**

6. Line 213-216, delete.

**Reply: Thank you for this suggestion, this has now been corrected in our manuscript.**

7. Table 2, Global warming, CO2, subscript.

**Reply: Thank you for pointing this out, this has now been corrected in our manuscript.**

8. Table 3, Delete "Results,".

**Reply: Thank you for this comment, this has now been corrected in our manuscript.**
